# From the Coast to the High Mountains: A Remote Sensing Survey of Disturbances and Threats to the Archaeology and Heritage of South Sinai

**DOI:** 10.1007/s11457-022-09335-2

**Published:** 2022-09-20

**Authors:** Sophie Tews, Letty ten Harkel, Ahmed Shams

**Affiliations:** 1 MarEA Project, School of Geography and Environmental Sciences, Ulster University, Coleraine, UK; 2 EAMENA Project, School of Archaeology, University of Oxford, Oxford, UK; 3 ICHM, Department of Archaeology, Durham University, Durham, UK

**Keywords:** South Sinai, Coastal archaeology/heritage, Cultural heritage management, Remote sensing

## Abstract

The archaeology and heritage of South Sinai is rich and varied. Most research to date has focused on the High Mountains, specifically the area around the famous St Catherine’s Monastery, placed on the UNESCO World Heritage List in 2002 (Saint Catherine Area, World Heritage Site 954). Recently, the Sinai Peninsula Research and the Endangered Archaeology in the Middle East and North Africa projects have focused on the landscape surrounding the St Catherine’s Monastery. These projects highlighted the wealth of archaeological and heritage sites spanning the prehistoric to modern periods, including sites that are still in use by local communities today, as well as the environmental and anthropogenic factors that threaten their survival, such as climate change, tourism, and the impact of infrastructure developments. By contrast, the archaeology and heritage of the coastal areas was never surveyed systematically until the research presented in this paper. Remote sensing work by the Maritime Endangered Archaeology project revealed a coastal landscape that is likewise rich in archaeological and heritage sites. As in the High Mountains, many of the coastal sites are under significant threat, but they do not enjoy the same level of recognition and protection. This paper compares the coastal sites to those in the High Mountains, including their disturbances and threats, and demonstrates the need for a locally specific heritage management and protection strategy for different parts of South Sinai.

## Introduction

**Fig. 1:**
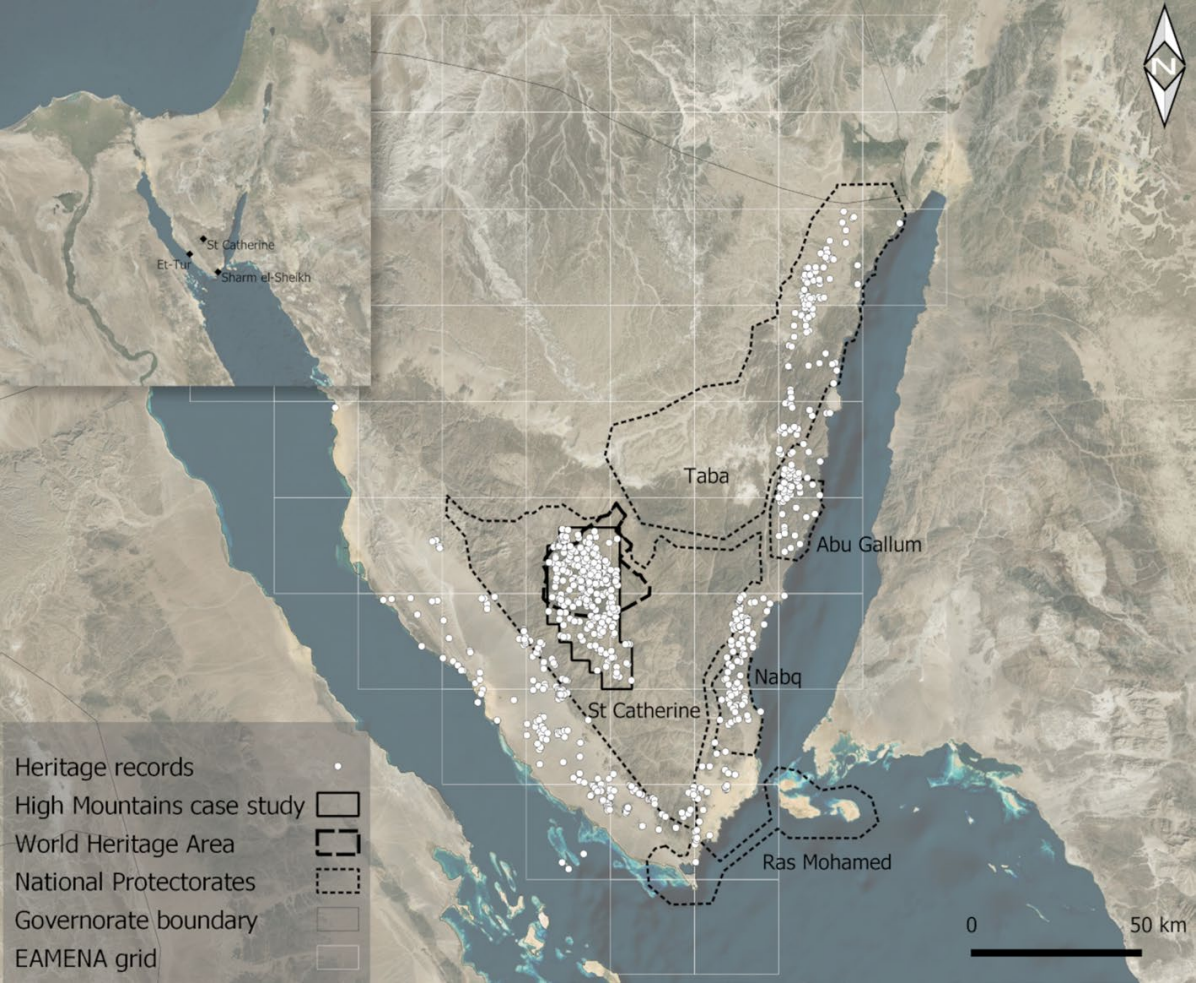
Location of study areas, heritage places and places mentioned in the text. Images generated in QGIS 3.16.4. Background imagery © Bing 2015, added through QGIS 3.16.4 XYZ Tile Layer functionality (http://ecn.t3.tiles.virtualearth.net/tiles/a{q}.jpeg?g=0&dir=dir_n') from https://github.com/nextgis/quickmapservices_contrib/tree/master/data_sources. Figure compiled in Gimp 2.10.22

The South Sinai Governorate covers the southern part of the Sinai Peninsula, bordered by the Gulf of Suez in the west and the Gulf of Aqaba in the east (Fig. 1). The peninsula takes up 6% of the total landmass of Egypt, but has 27.6% (700 km) of the country’s total coastline, giving it a relatively maritime character (Embabi 2020:506). It also has the highest variation in altitude of any region within Egypt, ranging from low-lying coastal plains to the High Mountains, whose highest peak—Jabal Katrin or Mount Catherine—reaches 2642 m above sea level. The region has many different natural environments that are all home to rare endemic species. On the coast, these include coral reefs and mangroves, while the arid inland regions consist of carbonate plateaus (the El-Tih and El-Egma plateaus) and rugged mountains dissected by deep canyon-like wadis, providing habitation areas for people and animals (the largely red granite High Mountains) (Grainger 2013:31; Embabi 2020:506; Omran and Negm 2020:27, 29)*.*

Archaeological evidence suggests that the peninsula functioned as a land bridge since prehistoric times, linking Africa and the Middle East (e.g. Rothenberg 1970:15; Grainger and Gilbert 2008:21). The High Mountains also play an important role in Islamic, Christian and Jewish belief, as Moses is believed to have received the Ten Commandments on top of Jabal Musa/Mount Sinai. The many archaeological and heritage sites in the High Mountains, mainly in the vicinity of Jabal Musa/Mount Sinai, were first mapped and studied by the British Ordnance Survey in the late nineteenth century (Wilson and Palmer 1869). Since then, South Sinai has seen repeated archaeological survey initiatives, often focusing on specific time periods and regions (e.g. Rothenberg 1970; Bar-Yosef 1982; Bar-Yosef et al. 1983; Phillips 1987; Eddy and Wendorf 1999; Mumford and Parcak 2003; Zalat and Gilbert 2008; Shams 2011, 2014). However, a comprehensive survey of archaeological and heritage sites in the vast coastal plains of the peninsula remained lacking until the research presented here.

Protection and conservation initiatives on the coast, especially along the Gulf of Aqaba, are mainly focused on natural heritage. South Sinai has five of Egypt’s 30 Natural Protectorates (Fig. 1), administered by the Egyptian Environmental Affairs Agency (EEAA) of the Ministry of Environment. Four of these—Taba, Abu Gallum, Nabq and Ras Mohammed—cover a large part of the eastern Sinai coastline (EEAA 2012; Leach et al. 2013). Part of the fifth—the Saint Catherine Protectorate, located inland—is also a UNESCO World Heritage Site. In total, these protected areas cover circa 11000 km^2^, which is over 40% of the peninsula (Gilbert 2013:43). In the official description on the EEAA’s website, cultural heritage is only mentioned in the context of two of these protectorates: those of Saint Catherine and of Taba (https://www.eeaa.gov.eg/en-us/topics/nature/protectorates/protectoratesdescription.aspx). Nevertheless, as we demonstrate here, many archaeological and heritage sites also exist within the coastal Protectorates of Abu Gallum, Nabq and Ras Mohammed, and along the southwestern coast (Fig. 1). What is more, despite the implementation of protection mechanisms, cultural heritage in the region is threatened by a range of interconnected natural and anthropogenic factors.

This paper presents the results from the Maritime Endangered Archaeology (MarEA) remote sensing survey of coastal sites in South Sinai, mainly based on remote sensing imagery in combination with published sources. MarEA’s results are compared to the results from the Endangered Archaeology in the Middle East and North Africa (EAMENA) and Sinai Peninsula Research (SPR) surveys, which focused on the High Mountains. After briefly discussing the range of methodologies that underpin the study, the paper will continue to present two case studies—one coastal and one inland—before describing in detail the results of analyses carried out with the joint EAMENA/MarEA database. A significant part of the paper is devoted to an in-depth overview of the range of threats and disturbances to the archaeology and heritage of South Sinai, concluding with a discussion of current protection policies as well as ways to enhance the protection of the sites.

In Egypt, the term ‘heritage’, rather than archaeology, tends to be used for remains that are less than 100 years old (Kenawi pers. comm.). This paper roughly follows the same distinction although ‘cultural heritage’ (and ‘heritage management’) is used more broadly to distinguish man-made features from ‘natural heritage’. ‘Heritage place’ is a specific database term to refer to an entry in the EAMENA database (usually a ‘site’, but it can also refer to a group of sites, an individual building, or even an artefact with spatial coordinates). Given the difficulty of dating man-made remains from remote sensing alone, however, often the distinction between ‘archaeology’ and ‘heritage’ is not clear-cut.

## Methodology: MarEA, EAMENA and SPR

The MarEA remote sensing survey covered South Sinai’s entire coastal stretch, from the international boundary between Eilat and Taba at the head of the Gulf of Aqaba in the east to the southern tip of the Suez Canal in the west. Results are compared to data from the better-researched High Mountains region, which includes the Saint Catherine World Heritage Area, generated by the EAMENA and Sinai Peninsula Research (SPR) projects. The ongoing MarEA and EAMENA projects rely on openly accessible satellite imagery, aerial photography and published documentation to identify archaeological and heritage sites in the MENA (Middle East and North Africa) region, assess their condition, and identify any threats (Bewley et al. 2016; Andreou et al. 2020). These sites or ‘heritage places’ are documented in an online database, hosted by the University of Oxford, along with an assessment of their condition, the type of damage and possible threats (https://eamena.org/database; Rayne et al. 2017:1; Zerbini 2018; Ten Harkel and Fisher 2021). The EAMENA/MarEA database covers the entire MENA region and serves as a starting point for more focused research and/or conservation projects. Collaboration between the UK team and MENA-based governments and archaeology and heritage specialists was intensified during a series of training courses organised by EAMENA, and—after the MarEA project was founded—in collaboration with MarEA (Hobson 2019; Ten Harkel et al*.* forthcoming).

For this paper, satellite imagery from Google Earth Pro (GE), dating to the 2000s to 2020s, was complemented by high-resolution (2–4ft) historical satellite imagery from the KH-9 Hexagon satellite system, dating to 1974. To fill the chronological gap between the 1974 KH-9 imagery and the modern GE imagery, medium-resolution (30 m) LandSat imagery covering the period 1985‒2020 was analysed in Google Earth Engine (GEE), mainly to highlight areas of agricultural intensification. Finally, data from published research reports and detailed ground surveys such as SPR were also entered in the database (e.g. Finkelstein 1985; Dahari 2000; Beit-Arieh 2003; Kawatoko 2005; Mumford 2006; Shams 2011; Shams and Ten Harkel 2019).

The SPR project undertook a detailed ground survey in the High Mountains over several seasons, mainly focusing on the identification and mapping of archaeological and heritage sites alongside contemporary landscape use (Shams 2011). The incorporation of these data in the EAMENA/MarEA database has produced differences in the respective datasets for the coastal and inland areas. Sites identified exclusively from remote sensing imagery are usually harder to identify and date, which means that more aspects of a larger percentage of coastal heritage places have been recorded as *unknown.* Effectively, this means that these sites *appear* to be archaeological based on analysis of satellite imagery, but uncertainties that can only be ‘solved’ when sites are visited on the ground remain, including information on function and dating. For that reason, this paper will not comment in detail on the distribution of site types or chronology, but focus predominantly on disturbances and threats, in particular those that impact on the landscape more broadly (and are thus easier to identify from remote sensing imagery).

## Case Studies

This section puts a spotlight on two case studies, al-Tur on the coast and the St Catherine’s Monastery and the town of St Catherine in the High Mountains. They were selected because they are both rich in archaeological and heritage sites that exist in close proximity to a quickly developing urban landscape. Additionally, their comparison provides an opportunity to study the effectiveness of protection mechanisms for cultural heritage, because St Catherine’s Monastery lies within both a Protectorate and a World Heritage Area, while the area around al-Tur is not a protected area—all registered archaeological sites are subject to ‘The Antiquities' Protection Law’, no. 117, 1983 and its amendments in 2010/18.

### al-Tur

**Fig. 2:**
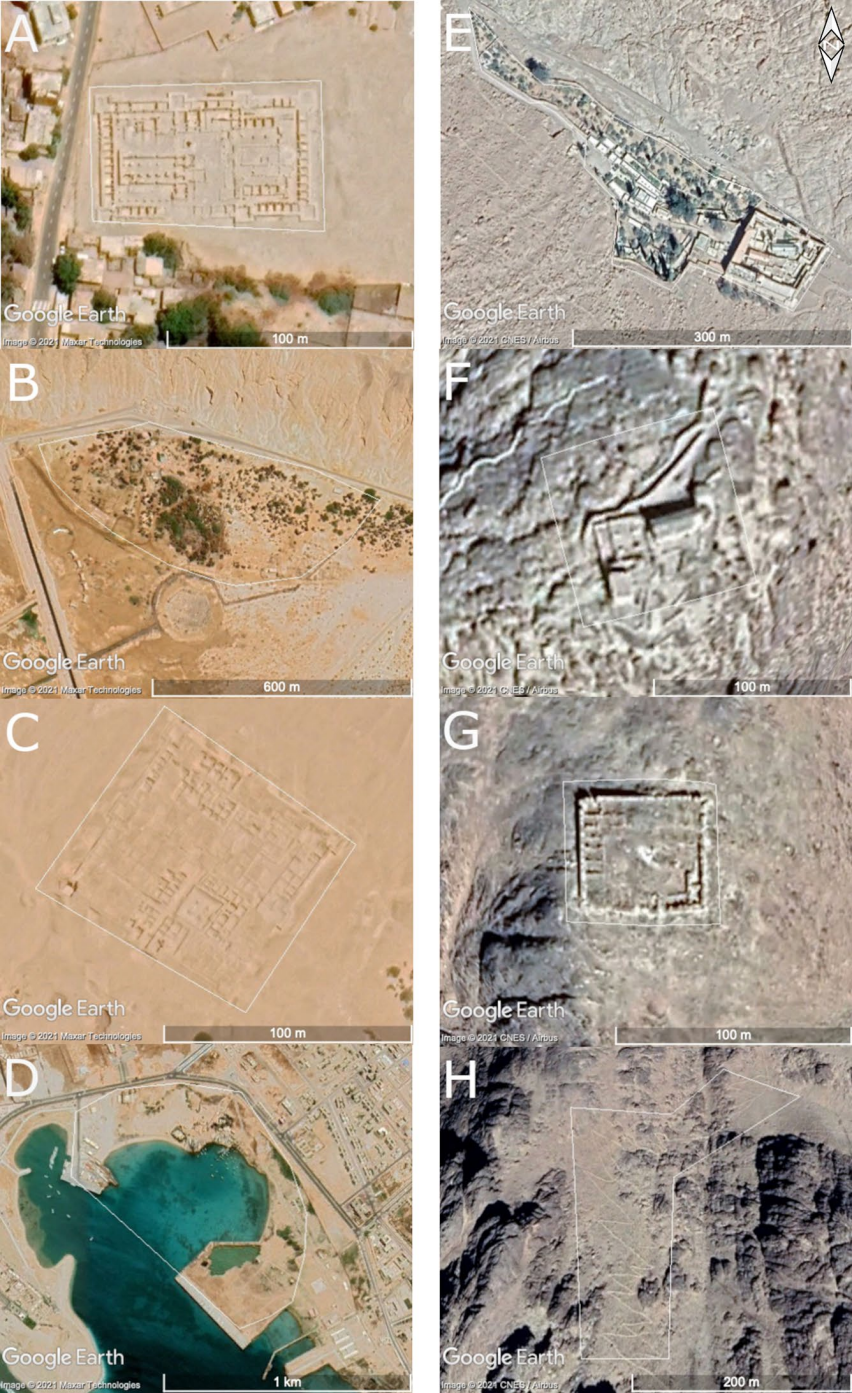
Examples of sites mentioned in the text, showing coastal sites on the left and sites in the High Mountains region on the right. **A**: EAMENA-0152822, Monastery of St John, al-Tur; **B**: EAMENA-0154388, Hamman Musa; **C**: EAMENA-0152819, Raya, al-Tur; **D**: EAMENA-0152821, al-Kilani medieval harbour; **E**: EAMENA-0116928, St Catherine’s Monastery with associated orchards; **F**: EAMENA-0134590, Musa/Chapel and Mosque of Moses; **G**: EAMENA-0134637, Qasr Abbas Basha/unfinished palace of Abbas Pasha; **H**: EAMENA-0135524, part of the footpath leading from Arba‘ein/Convent of the Forty Martyrs. Imagery from Google Earth Pro. Figure compiled in Gimp 2.10.22

Population levels in the Sinai have never been high, but even so, the peninsula hosts a variety of archaeological sites. These include prehistoric enclosures (Bar-Yosef 1982; Beit-Arieh 1986), dynastic anchorage points and mining sites, such as Tell Ras Budran (Mumford and Parcak 2003; Mumford 2006), and medieval fortresses at Nuweiba Tarabin and al-Tur (Pradines 2016). al-Tur will serve as our first case study, because of its rich history, connectivity to other places in the peninsula and beyond, and the urban development that poses a direct threat to the site.

al-Tur—ancient Raithou—is one of the few towns in South Sinai which does not rely solely on the tourist industry, like many of the towns on the eastern coastline. As capital of the South Sinai Governorate, al-Tur primarily functions as an administrative and industrial centre. Archaeological remains testify to its long history as a harbour on the Gulf of Suez, facilitating trade and accommodating travellers (Shuqair 1917; Kawatoko 2005). From the fourth century CE, the coastal area functioned as a monastic centre, with hermits living in caves in the Jebel Naqus mountain range north of the town. In the sixth century, Emperor Justinian commissioned the building of St John’s Monastery—one of three major monastic centres in South Sinai, with close links to the St Catherine’s Monastery in the High Mountains—to protect the monks and safeguard the trade route, highlighting its strategic position (Fig. 2a) (Finkelstein 1985; Dahari 2000). This monastery was in use until the twelfth century, after which it functioned as a Christian graveyard. The ruins of the monastic complex are still present in the landscape. Close to the monastery are hot springs, known as Hammam Musa, where Moses and the Israelites allegedly stopped on their journey through the Sinai (Fig. 2b), and which are now primarily functioning as a tourist attraction and a health resort.

Contemporaneously with the monastery, a medieval port was situated approximately 8 km south of the modern town of al-Tur. This walled town, Raya (Fig. 2c), also dates to ca. the sixth century and was built on a gentle slope overlooking the coastline. A regular street pattern divided the fort into five or six quarters with residential spaces, shops and storerooms, and one of the oldest mosques on the peninsula (Kawatoko 2005:851–852). More structures, presumably warehouses, more residential quarters and some public buildings, were located extramurally, in the coastal plain closer to the present coastline.

After the abandonment of the fort at Raya in the twelfth century, in the fourteenth century CE a new port was built in the al-Kilani area (situated inside the modern town) (Fig. 2d). Most of the buildings were constructed of coral blocks, similar to the buildings in the Raya fort. Although St John’s Monastery had been abandoned by the time this port was built, the town still had strong connections to the St Catherine’s Monastery; additionally, it served as a gateway and as a place of quarantine for Muslims returning from *hajj* (Shuqair 1917). Besides locally produced artefacts, archaeological investigations revealed artefacts from Syria, Palestine, the Arabian Peninsula, India, south-east Asia and Europe, indicating wide-ranging (trade) connections (Kawatoko 2005:854).

The clear artefactual evidence for connectivity to the wider Red Sea area is underlined by the morphology of the ports, especially that of Raya, which strongly resembles the morphology of other ports located on the Red Sea, such as Suakin and ‘Aydhab. These ports all started to emerge following the rise of Islam and subsequent Arab expansion. They functioned to facilitate trade from the Indian Ocean to the Mediterranean and pilgrim traffic associated with the *hajj* (Breen 2013). The fact that Raya was a walled fort indicates that this was not merely a stopover place. Clearly, a big investment was made to safeguard this site. Together with the ports at Nuweiba Tarabin and Aqaba, both on the Gulf of Aqaba, al-Tur functioned as maritime caravanserai, defended freshwater sources and ensured safe passage of ships (Pradines 2016:83). These ports, in turn, were part of a bigger network of port sites and cities that existed throughout Egypt between the ninth and early nineteenth centuries (Pradines 2016). Excavations at both Raya and al-Kilani, carried out by a Japanese-Egyptian team (Kawatoko 2005; Kawatoko and Tokunaga 2006), focused primarily on the architectural remains, however, which means that details about their socio-economic organisation, relation to their hinterlands and their long-distance connections to these other sites and the wider Red Sea area, remain elusive. The expeditions also did not take into account any archaeological remains that might be located underwater. From the satellite imagery, it appears that there are no jetties, quays or piers, which would mean that boats were beached, which was not uncommon for harbours of this kind (Breen 2013:318).

Since the 1970s, the abundance of oil fields in proximity to the town has transformed al-Tur from a small fishing hamlet to a transit town. In 2018, a new road was constructed through the El Qa’a Plain as part of the Sinai Development Programme, connecting Suez to Sharm el-Sheikh and passing al-Tur. This has given the development of the town and the surrounding coastal landscape between the sea and the High Mountains a new impetus.

### The High Mountains

St Catherine’s Monastery is a Byzantine Orthodox monastery, founded in the sixth century CE at the foot of Jabal Musa/Mount Sinai (Fig. 2). The site has been continuously occupied since at least the fourth century CE and is ‘the oldest Christian monastery still in use for its initial function’ (Shackley 1998:124). The monastery defences, like the Monastery of St John in al-Tur built at the order of the Emperor Justinian, comprise granite walls up to 20 m high and 2‒3 m in thickness, inside of which is a maze of buildings including 12 chapels, living quarters, an icon gallery, a hospice, a refectory and multiple ancillary buildings (Shackley 1998:124–25). The whole area is sacred to Muslims, Christians and Jews and contains numerous other archaeological and religious sites that bear testimony to the importance of the monastery. These include various places for worship as well as the unfinished summer palace of Abbas Pasha, the Khedive of Egypt, connected by ancient paths (Fig. 2), many of which were visited during the SPR project (Shackley 1998:124; Shams 2011). Once the road to the monastery was tarmacked in the 1980s, visitor numbers grew exponentially, as did the town of St Catherine to the north-west, and the need to protect the area’s natural and cultural heritage.

The first initiative that was launched to protect the area was the formation of the St Catherine Protectorate (Fig. 1), the only protectorate in Egypt with a relatively sizable population and urban development (Grainger and Gilbert 2008). Designated as a Protectorate in 1988 under the management of the Egyptian Environmental Affairs Agency, its boundaries were formally defined in 1996 as part of an EU-funded project (Gilbert 2010:101). It was designed ‘to encourage sustainable patterns of tourism and to protect natural habitats in this sensitive environment’ (Hobbs et al. 1998:238) along with the archaeological and religious sites in the region, and to involve and support the local Bedouin population (Hobbs et al. 1998; Gilbert 2010:101‒2, 104‒9).

In 2003, as EU-funding for the Protectorate ceased, the central part of the area had been added to the World Heritage List as the Saint Catherine Area (WH Site 954) since 2002 (Fig. 1), but problems were becoming apparent (Gilbert 2010:109). Although the Egyptian government continued to allocate substantial resources to promote eco-tourism, a 2007 evaluation (Paleczny et al. 2007; discussed in Gilbert 2010:109‒10; also see Fouda et al. 2006) highlights a range of issues caused by underfunding, staff shortages and bureaucratic complications, including poor coordination between different levels of management and often-competing development and legal mandates. Amara (2017:188‒89) has furthermore drawn attention to the fact that travel companies engage insufficiently with environmental issues. Tensions between the Bedouin and the Egyptian government and developers have risen because of the differential enforcement of the Protectorate’s rules (Gilbert 2010:114; also see Amara 2017:188‒89). In the face of these changes, the SPR project—recently in collaboration with the EAMENA project—has drawn attention to the importance of the ‘living’ heritage of the area, in particular the traditional Bedouin agriculture comprising orchards and water management systems, which do not enjoy the same levels of protection as registered archaeological and religious sites (e.g. Shams 2014, 2021; Shams and Ten Harkel 2019).

The condition of the World Heritage Area has reportedly been relatively stable compared to surrounding areas, but the situation is nevertheless far from ideal (Paleczny et al. 2007:6‒8; Grainger and Gilbert 2008:32). Official plans for the site are frequently ignored and UNESCO World Heritage conditions violated by installing street lights, uncontrolled residential development outside the development zone and planting exotic plants, which causes radical change to the original landscape (Paleczny et al. 2007:6‒8; Gilbert 2010:114). The impact of tourism is enormous and growing. In 1998, 97,000 annual visitors were recorded, which represented a 300% increase over the decade leading up to it, and numbers kept growing (Shackley 1998:124, 129; Karima 2017; Tomazos 2017). Although the events following the Arab Spring, the 2015 Sinai plane crash and more recently the global coronavirus pandemic have interrupted the upward curve, more growth is forecast and further development to accommodate mass-tourism to the area is taking place (Shams and Ten Harkel 2021).

## Results

For the analyses presented in this paper, a total of 793 heritage places were included, all of which were recorded by the MarEA and EAMENA projects (the latter incorporating 121 sites surveyed by SPR). Of these, 483 are on the coast and 310 in the High Mountains case study area. In addition to the location of the sites, their function, features, overall condition, disturbances and possible threats were all documented. The research highlighted functional differences between the coastal areas and the High Mountains, as well as some differences in the frequency of disturbances and threats, emphasising the unique character of both areas and reinforcing the need to develop tailored measures to protect Sinai’s unique coastal areas. Although it must be acknowledged that data obtained by remote sensing techniques are unavoidably less detailed than data collected in the field, the database nevertheless provides a decent overview of the relative density of archaeological and heritage sites in different areas of the Sinai Peninsula (Fig. 1). Furthermore, data gathering through remote sensing has the great advantage that vast areas can be covered in a relatively short time, enabling comparisons between different regions and landscapes (also see Ten Harkel et al. 2021).

### Function

**Fig. 3:**
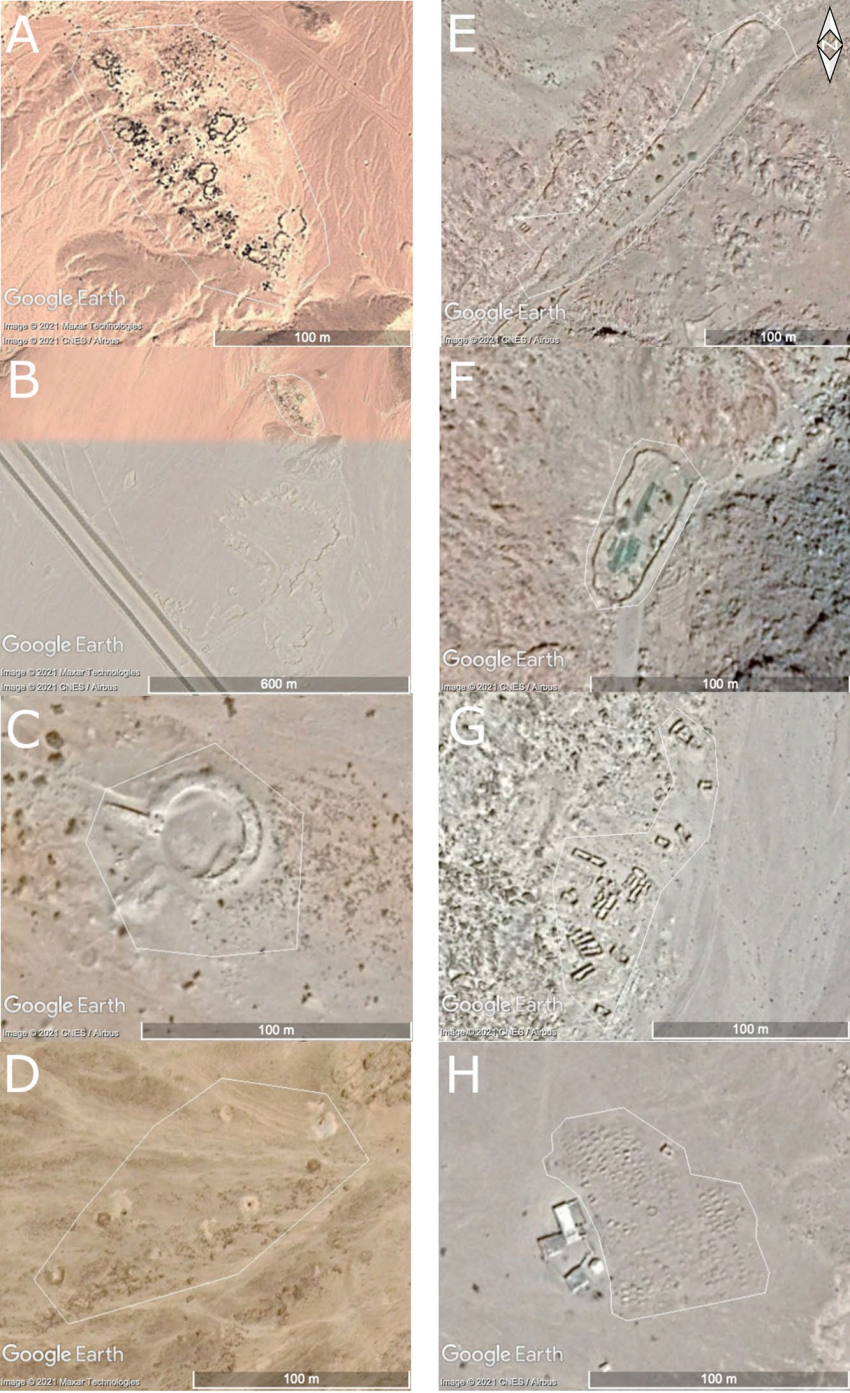
Examples of site functions and some disturbances/threats, showing coastal sites on the left and sites in the High Mountains region on the right. **A**: EAMENA-0152919, an *agricultural/pastoral* site; **B**: EAMENA-0152919, the same *agricultural/pastoral* site zoomed out, showing the threats of road construction and bulldozing (the colour difference of the background is due to the transition between different satellite images of different dates); **C**: EAMENA-0182335, Tell Ras Budran, an example of an *infrastructure/transport* site; **D**: EAMENA-0158970, an example of a likely prehistoric *funerary/memorial* site seemingly disturbed by looting pits; **E**: EAMENA-0182281, an *agricultural/pastoral* site, likely disturbed by *water damage* (flash floods); **F**: EAMENA-0135073, an *agricultural/pastoral* site, still in use (*occupation/continued use* is also recorded as a disturbance in the database, but the impact is usually low); **G**: EAMENA-0135479, an example of a *domestic* site; **H**: EAMENA-0134649, an Islamic-period cemetery and example of a *funerary/memorial* site, which is also probably still in use. Imagery from Google Earth Pro. Figure compiled in Gimp 2.10.22

**Fig. 4:**
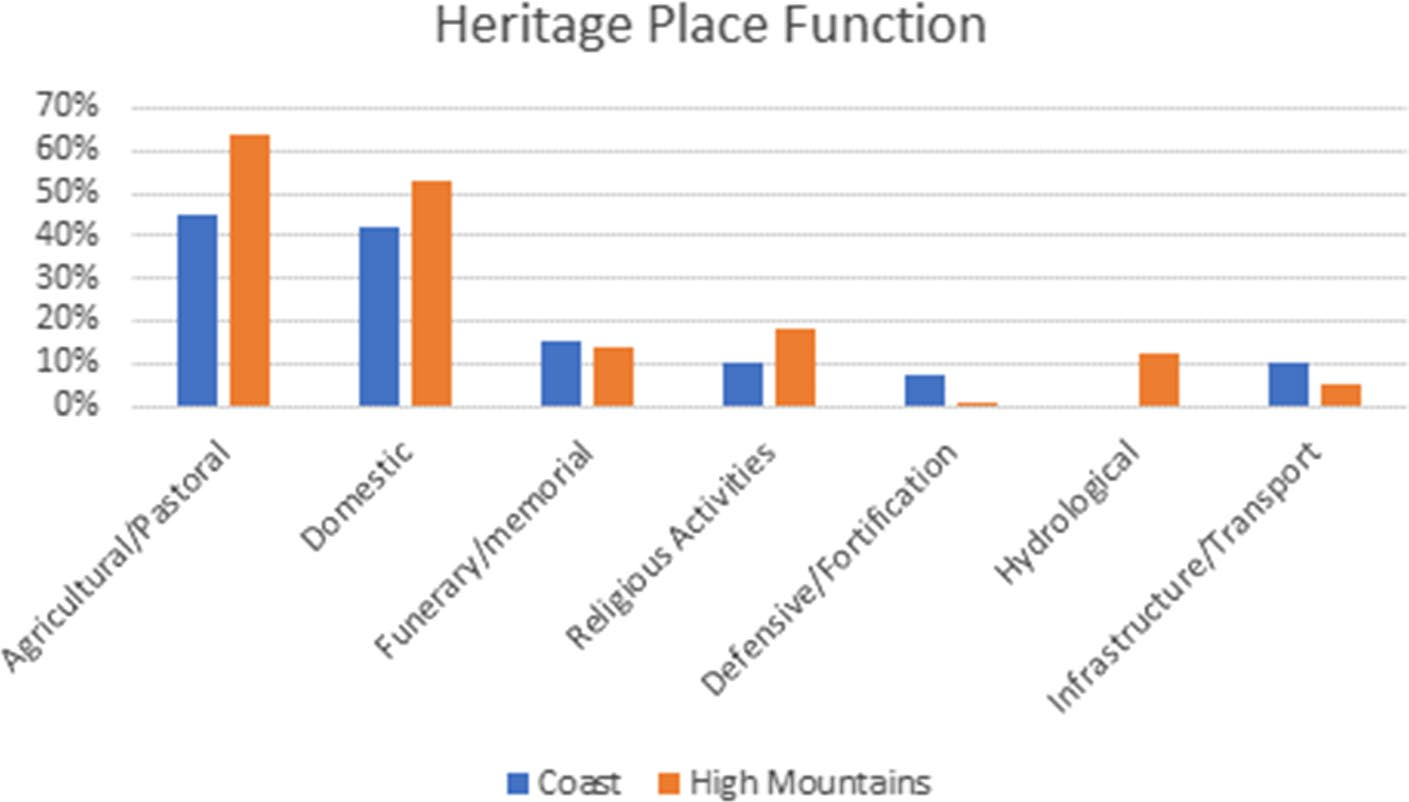
The most common heritage place functions as documented in the EAMENA/MarEA database

The recorded functions for the heritage places include the categories *agricultural/pastoral* (e.g. Figure 2e, Fig. 3a, e, f)*, domestic* (e.g. Figure 2a, c, e, Fig. 3g), *funerary/memorial* (e.g. Figure 3d, h), *religious* (e.g. Figure 2a, e, f), *defensive/fortification* (e.g. Figure 2d, g), *hydrological, infrastructure/transport* (e.g. Figure 2h, Fig. 3c) and *hunting/fishing*, as well as *unknown*. Largely as a result of the inclusion of the SPR ground survey data, site functions could be assigned to the sites in the High Mountains case study area with more certainty (95% of the heritage places had one or more functions assigned to them) than to the sites in the coastal zone (only 13% of the heritage places had an assigned function).^1^ However, if we leave out the sites with an *unknown* function, several clear differences between the sites in the coastal zone and the High Mountains remain (Fig. 4). These include a higher percentage of religious (18%) and hydrological (12%) sites in the High Mountains area compared to the coastal zones (10% and absent, respectively). This difference results from the inclusion of the central part of the Saint Catherine Area within the study area, as well as the importance of water management systems for irrigation in this mountainous agricultural landscape. Furthermore, in the coastal zone 7% of the sites had the function *defensive/fortification* attributed to them, whereas this category only makes up 1% of the heritage places in the High Mountains. Also, the number of heritage places with an *infrastructure/transport* function is higher in the coastal zone (10%, compared to only 5% in the High Mountains). Finally, all the sites that have *trade/commercial* as a function (less than 1% in total) are in the coastal zone.^2^ This points to functional differences between the High Mountains and coastal areas, highlighting the significance of the coastal region for connectivity and transportation.

### Condition

**Fig. 5:**
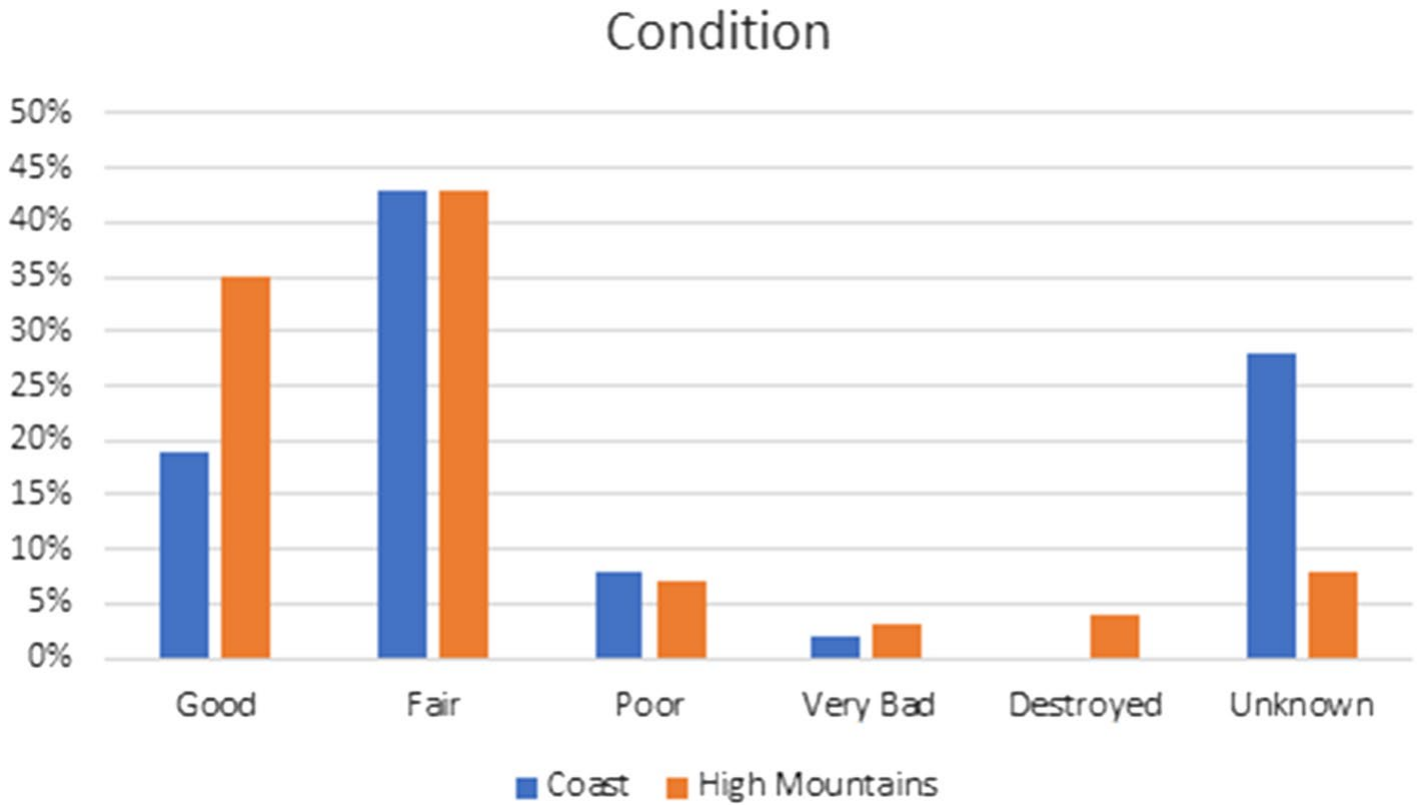
Heritage place condition as documented in EAMENA database

Site condition can be documented in the EAMENA/MarEA database ranging from *good* (virtually no evidence of active deterioration and appears to be structurally stable) to *destroyed* (a site has been impacted severely by anthropogenic and/or natural causes, and no longer retains integrity or sound archaeological data) (Fig. 5). In both the coastal zone and the High Mountains case study area, 43% of heritage records were considered to be in *fair* condition (shows little evidence of active deterioration and appears stable), while only a small percentage of heritage records in both areas were recorded as being in *poor* (moderate signs of deterioration and structural instability) or *very bad* (serious signs of active deterioration and severe structural instability) condition. In the coastal zone, no sites were labelled as *destroyed*; in the High Mountains case study area, destroyed sites clustered in the town of St Catherine, where extensive urbanisation since the late 1970s and early 1980s—observable through comparison of the 1974 KH-9 imagery with modern GE imagery—has caused significant landscape change (also see Shams and Ten Harkel 2021). For 28% of the heritage places in the coastal zone and 9% of the heritage places in the High Mountains, a condition assessment could not be made, highlighting the importance of more targeted ground survey, especially in the coastal region.

### Disturbances and Threats to Identified Heritage Places

**Fig. 6:**
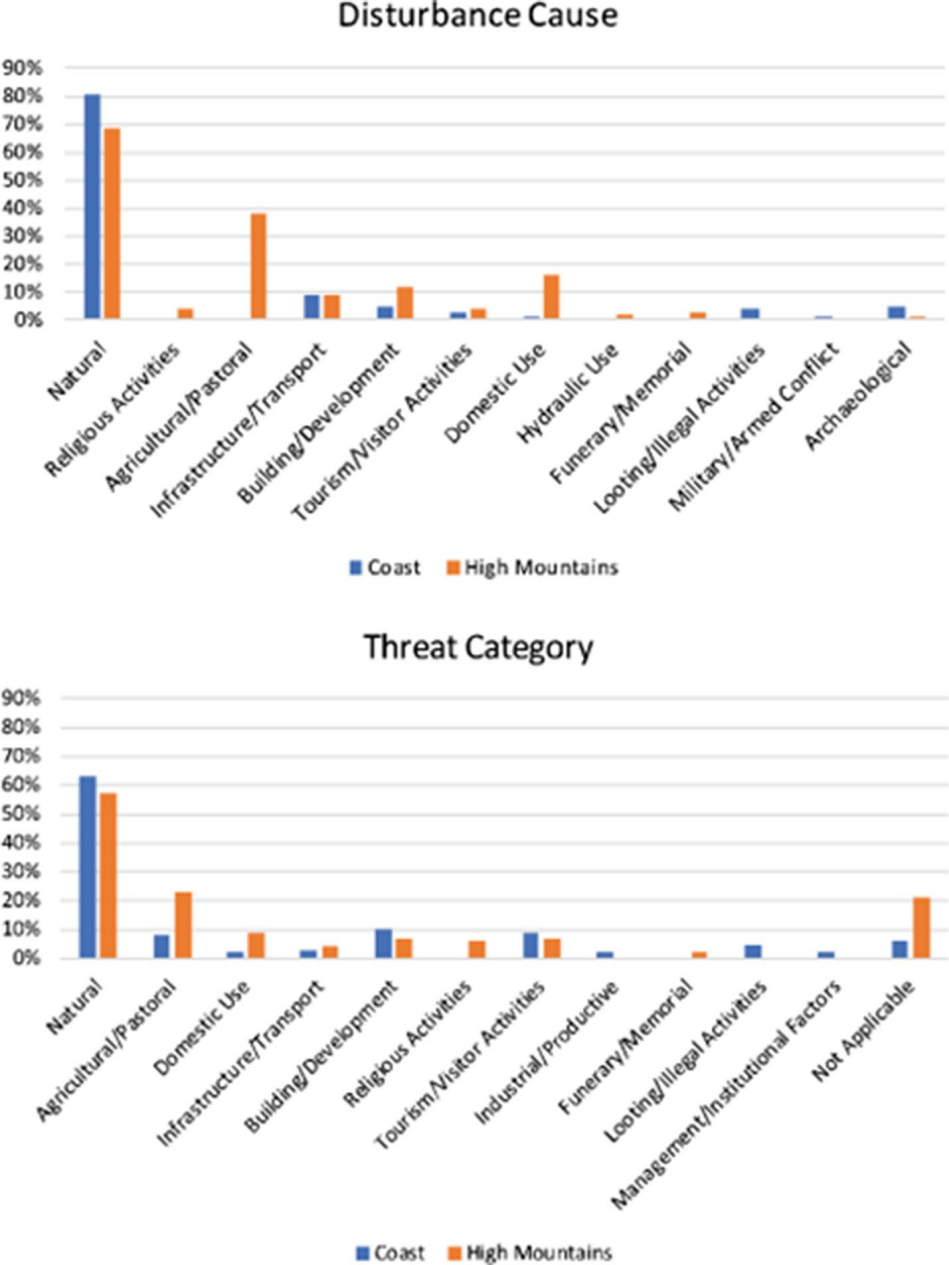
Most frequently occurring disturbance and threat causes

In the EAMENA/MarEA database, disturbances are developments that have impacted directly on a heritage place, while threats are developments in the vicinity of a heritage place that may become disturbances in the near future (e.g. Fig. 3b).^3^ During the analyses underlying this paper, a disturbance cause could be identified in 138 (31%) heritage places in the coastal zone, and in 186 (60%) of the heritage places in the High Mountains, again highlighting the need for more detailed investigation of the coastal heritage and archaeology (Fig. 6). These disturbance causes are for a large part interconnected, and some significant similarities and differences can be deduced from the two datasets. The most frequently occurring disturbances, which will be discussed in more detail below, are *natural*, *building and development* and *infrastructure/transport*. A significant difference between the two regions is the relative impact of disturbances caused by *agriculture/pastoral* activity and *domestic use*. In this particular dataset, these disturbance causes primarily represent the continued use of historical orchards, field systems and associated buildings, which makes up a large but low-impact disturbance category in the High Mountains region, but is virtually absent for the heritage places in the coastal zone. To the contrary, based on the analyses presented here, archaeological excavation and illegal activities both seem to occur in the coastal zone, mostly on the unprotected west coast, but less so in the High Mountains.

#### Natural

Climate change has been identified as a major threat to the Sinai Peninsula (Dadamouny and Schnittler 2016). The increase in greenhouse gases and melting of Arctic sea ice are linked to a rise in average global temperatures, causing a rise in sea levels and an increase in storm surges and other extreme weather events. This particularly affects the sensitive coastal areas and tourist cities such as Sharm el-Sheikh, al-Tur and St Catherine (Dadamouny and Schnittler 2016; Raey and Askari 2020:399‒418; Shokr 2020:100). Since the 1970s, increasing temperatures and decreasing rainfall have led to severe droughts, interrupted by unpredictable rainfall in high volumes and flash floods (Grainger and Gilbert 2008:32; Dadamouny and Schnittler 2016). This affects both the High Mountains, dissected by a network of wadis where human occupation is concentrated, and the coastal regions along the Gulf of Aqaba and the Gulf of Suez, where the wadis drain, at times causing severe infrastructure damage and loss of life (Vanderkrimpen et al. 2010; Youssef et al. 2011:615; Cools et al. 2012; Embabi 2020:506; Raey and Askari 2020:430).

Unsurprisingly, *natural* causes were identified as the most common disturbance and threat to the heritage places of the region, both for coastal and inland areas. Having said that, direct evidence for global sea level rise in the shape of actual flooding of coastal sites has not (yet) been identified: instead, most disturbances comprise the effects of *water and/or wind action* causing *erosion/deterioration* or sometimes *collapse/structural damage* to (parts of) heritage places (e.g. Fig. 3e). This is not exclusively a result of climate change, however, as anthropogenic factors including unplanned and rapid settlement development and construction as well as major changes in land use patterns also contribute to the likelihood of these disturbances (Youssef et al. 2011:612). It may be relevant to point out that on a national level—based on the 2011 Food and Agriculture Organisation (FAO) combined assessment of average temperatures, poverty levels and community resilience—South Sinai was considered to have relatively high agricultural resilience to climate change (Raey and Askari 2020:417, Fig. 14.16). This, in turn, may have contributed to the governmental decision to resettle people from other parts of Egypt to the Sinai, including the coastal plain of al-Tur, resulting in additional disturbances and threats to heritage places (Gilbert 2013:46; Al-Monitor 2021).

#### Urban and Infrastructural Development

In the harsh environment of the Sinai Peninsula, habitation concentrates in a few suitable places, as it has done throughout history. The towns of South Sinai are located mainly on the coast, an exception being the town of St Catherine in the High Mountains. They have been subject to urbanisation that has intensified since the Israeli occupation in the late 1960s and 1970s, when the traditional way of semi-nomadic life of the Bedouin slowly started to shift towards a settled life. New economic activities were introduced, linked to industry (e.g. oil extraction in the Gulf of Suez) and market economy, such as leisure tourism on the Gulf of Aqaba and eco-cultural tourism inland, especially in the vicinity of Mount Sinai (Lavie 1991; Hobbs 1995). These developments are intensified by the large resettlement scheme initiated by the Egyptian government, aiming to transform large parts of the Sinai—traditionally a periphery area with a desert culture and history—into a region integrated with a nationwide development strategy, where Nile Delta Egyptians gladly settle. Although this scheme has not been without drawbacks (Gilbert 2013:46), large-scale extension and improvement in the peninsula’s infrastructure is currently underway, and new houses, schools, hospitals, and shopping centres are being constructed, while agricultural exploitation is increased to support the growing population.

**Fig. 7:**
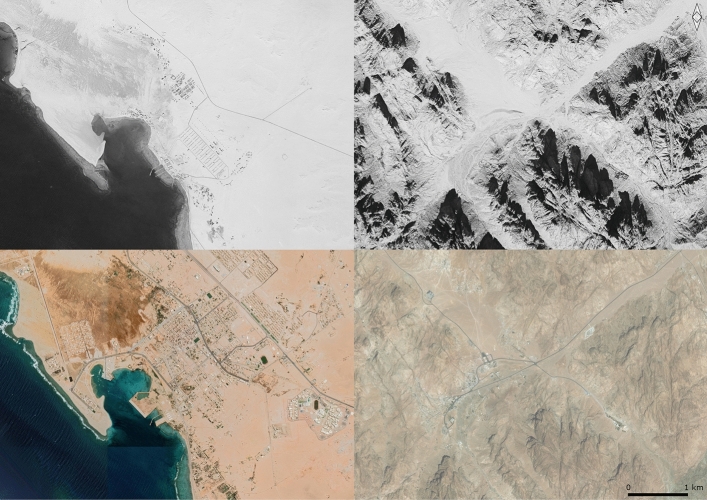
al-Tur (left) and St Catherine (right) areas in 1974 (KH-9) (top) and 2010s (Google Earth) (bottom), showing the scale of urban development. Images generated in QGIS 3.16.4. Google Earth imagery © Google Earth 2015, added through QGIS 3.16.4 XYZ Tile Layer functionality (https://mt1.google.com/vt/lyrs=s&x={x}&y={y}&z={z}) from https://github.com/nextgis/quickmapservices_contrib/tree/master/data_sources. KH-9 imagery available from the USGS, georeferenced in QGIS 3.16.4 using the georeferencing tool. Figure compiled in Gimp 2.10.22

The process of urbanisation is clearly visible from satellite imagery. The KH-9 imagery from al-Tur dated to 1974 shows the town as a small fishing hamlet, centred around the fourteenth to twentieth-century al-Kilani area. Recent Google Earth imagery shows that the town, with both residential and agricultural areas, has extended towards the northeast (Fig. 7). Besides the obvious damage and destruction caused by construction close to archaeological and heritage sites, there are several additional effects. One of these is the extension of the waterfront in the old harbour, where traditional fishing vessels have been replaced by motorised boats. Although it is unclear whether there is any underwater archaeology at this site, motorised boats could possibly have a more damaging effect on any submersed archaeological remains than the traditional boats. Similarly, although oil spillages from both on-shore and off-shore oil extraction form a major threat to the maritime environment (Hereher 2013:1476), any effects on underwater archaeology caused by oil platforms and pipes in the Gulf of Suez and large vessels heading for the Suez Canal and the commercial port at Aqaba are poorly investigated and remain largely unknown. Another effect of the modern development of the town is that the traditional coral buildings in this area are increasingly being abandoned and falling into ruin (Nawata et al. 2012).

In some cases, development activities have a positive effect on the protection of cultural heritage, at least initially. The development of a hotel strip south of al-Tur was cancelled after the discovery of the Raya fort and the designation of the area as a district for the preservation of cultural properties (Prime Minister’s order, no 3340, November 2, 1999). As a result, urban development does not pose a direct threat to this site now; however, this does not equal sufficient protection. The site was excavated over twenty years ago, but the trenches were never completely backfilled; the same goes for the Monastery of St John. Consequently, the fragile architecture of these sites lies exposed to the weather and unregulated human intervention, and is polluted by rubbish.

In the High Mountains, urbanisation is concentrated in the heart of the St Catherine World Heritage Area (Fig. 7). The exponential growth of the town of St Catherine—the fastest-growing town in Egypt—is a direct result of increasing tourism, discussed in more detail in the next section. The situation for the Sinai coast is more complex, especially along the Gulf of Suez: in addition to tourism, more factors including industrialisation contribute to rapid development. Once more, this emphasises the need to develop a more comprehensive heritage management strategy for the coastal areas that considers their unique and complex requirements.

#### Mass Tourism

South Sinai is a major tourist destination, appealing to mostly European and Israeli tourists for the combination of beach and scuba diving holidays with desert scenery and Bedouin culture, as well as religious tourism/pilgrimage to St Catherine’s Monastery (Shackley 1999:544; Grainger 2003:33; Uriely et al. 2009). The history of coastal tourism in the Sinai has taken off exponentially since the 1980s. The first small-scale tourism was established in Nuweiba, Dahab and Sharm el-Sheikh along the Gulf of Aqaba by the Israelis during their occupation of the peninsula in the late 1960s and 1970s (Hawkins and Roberts 1994:504). After the Israeli withdrawal in 1982, the Egyptian government developed this further in tandem with the establishment of a strong security presence, combining economic and national interests (Gilbert 2013:46). Since the 1980s, vast stretches of the eastern coast of the peninsula—outside the designated series of Natural Protectorates and despite the entire coast itself being protected—has been transformed into an almost completely serried strip of hotels and resorts.

Tourism has a far-reaching effect as a catalyst for infrastructure development and over-exploitation of natural resources. Examples include shoreline erosion, irrational land use, overgrazing and over-cutting of plant resources, leading to a decrease in biodiversity (Shokr 2020:112; Shaltout et al. 2021; but see Gilbert 2010:121‒22). Concerns for this amount of construction so close to the shore with delicate coral reefs has often been expressed and studied (e.g. Hawkins and Roberts 1994; Abdel-Kader et al. 1998; Mona 2019), but this is not so much the case for effects on the cultural heritage of the peninsula.

Travel to the High Mountains for religious purposes has a longer history, although it has intensified and diversified since the late nineteenth and early twentieth centuries, when technological developments made travel to remote areas easier. From this period onwards, the area was also increasingly visited for its natural beauty and hunting possibilities (Manginis 2016; Abdel Rahman, 2017; Ten Harkel et al. 2018). Travel nonetheless remained relatively difficult until the 1980s, when the road network (mostly dirt-tracks) leading into the interior of South Sinai was tarmacked and coachloads full of tourists were able to visit the monastery on a regular basis (Ten Harkel et al. 2018). This process will now be intensified as a result of the so-called ‘Great Transfiguration over the Land of Peace project’, which will see the imposition of an extensive infrastructure including a road network and hotels, aimed to accommodate mass tourism to the area that will practically double the size of the built-up area near the town of St Catherine (Ten Harkel et al. 2018; Ahram Online 2020; Emam 2020; Shams and Ten Harkel 2021).

*Tourism/visitor activities* have been recorded as a disturbance cause for 3% (coast) and 4% (High Mountains) of the documented heritage places, and as a threat for 9% (coast) and 7% (High Mountains) of the heritage places. The direct effects of current *tourism/visitor activities* on the heritage places in the EAMENA/MarEA database are relatively minor, but the planned *tourism/visitor activities* that aim to attract mass numbers will have a far-reaching impact on visitor experience and the historic character of the landscape. In addition, they will result in more visitors to currently remote areas along the coast and across the mountain range. Thus, it is the wider impact on the region that forms a case for concern, including the rapid urbanisation of the coastline and the town of St Catherine—located inside the World Heritage Area—and related processes such as population growth and agricultural expansion.

#### Agriculture

Another disturbance, noted primarily for the High Mountains case study area (38%), is *agricultural/pastoral* activity. This is only recorded in the EAMENA/MarEA database where it impacts *directly* on recorded heritage places. In the High Mountains, this usually takes the shape of the *occupation/continued use* of historical orchards, a type of ‘disturbance’ that has little to no negative impact on the heritage sites: on the contrary, it helps to preserve them (e.g. Fig. 3f). Variations in the severity of disturbances are important when informing management practices, and the EAMENA/MarEA database has functionality built in to record the relative severity of disturbance impact. In this context, it is relevant to refer to the continuing emphasis by some academics and the Protected Area Management Unit on ‘Bedouin overgrazing’, discussed by Gilbert (2010:121‒36), as the main negative factor influencing biodiversity, despite growing evidence that a range of human and ecological factors, including reduced rainfall, are to blame as much if not more so. Gilbert (2010:121‒36) argues that this is in part a result of negative stereotyping of traditional Bedouin lifestyles.

The impact of agricultural expansion on the coastal zone is more extensive, and linked to population growth and increased urbanisation. In the al-Tur and High Mountain areas, the main sources of water are (seasonal) precipitation and low-salinity groundwater. During the last decades, this was more than was needed for the local population, and excess was exported to nearby regions such as Sharm el-Sheikh and Abu Rudeis, although desalination is widely used along the Gulf of Aqaba as well (Rayan et al. 2001:75‒76, 78). In both al-Tur and Sharm el-Sheikh, this has led to a huge expansion of agricultural activities.

**Fig. 8:**
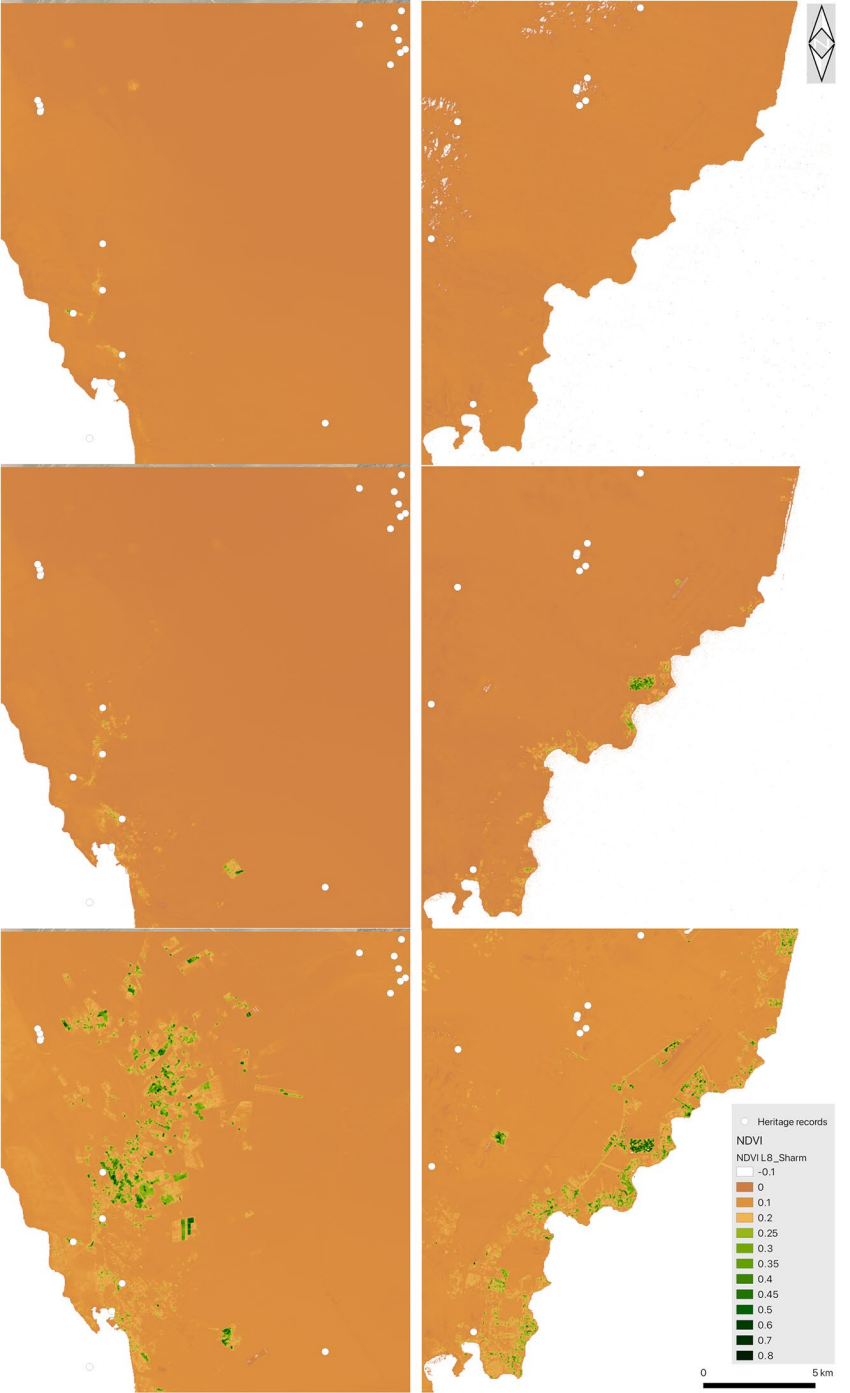
On the left, the al-Tur area in 1985, 2000 and 2020. On the right, the Sharm el-Sheikh area in 1985, 2000 and 2020. The dark/green areas are areas of increased vegetation representing agriculture. (The pale speckling in the top right image are probably clouds.) White dots are heritage places recorded in the EAMENA/MarEA database. Images generated from LandSat 5, 7 and 8 imagery in Google Earth Engine and QGIS 3.16.4. Figure compiled in Gimp 2.10.22

To monitor these changes, we analysed LandSat imagery for the period 1985–2020 in Google Earth Engine (GEE), mapping the normalised difference vegetation index (NDVI)—a remote sensing index for healthy green vegetation, computed as the difference between near-infrared (NIR) and red (RED) reflectance divided by their sum—over time. Figure 8 shows the results, highlighting the major increase in vegetation representing agriculture, especially in the last 20 years. Although this expanse in cultivation has not impacted directly on recorded heritage places, the impact on the historic landscape is nevertheless apparent.

#### Looting and Illegal Activities

Satellite imagery does not offer sufficient information to pinpoint *looting* or *illegal activities* with certainty. However, several (4%) heritage places documented in the coastal zone show features that could be interpreted as evidence for looting, mainly consisting of pits in and around the heritage place, for example on site EAMENA-0158970, where several pits seem to be dug in and around prehistoric tombs (Fig. 3d). In addition to disturbances caused by possible looting, for 5% of the heritage places in the coastal zone, looting and other illegal activities were recorded as a possible threat. However, perhaps a bigger threat to much of the archaeological and heritage sites is appropriation or land grabbing. This tends to happen when land, in this case owned by the Ministry of Antiquities, is or seems to be outside the continuous monitoring by the government (Ikram 2013:367). There is no clear evidence that this is already happening, but appropriation could nevertheless be a threat to some areas of al-Tur, including the old buildings at al-Kilani, now an abandoned area. Under Egyptian law, buildings that are more than 100 years old are considered historic monuments, even if they are unregistered with the antiquities authorities (Ikram 2013:367). As the buildings at al-Kilani are ruins, however, their demolition could provide space for other purposes, a well-known risk in urban contexts (Ikram 2013). Although owners can be penalised, much information is lost forever once the original buildings are destroyed.

In order to identify potential locations to loot, it has been suggested that organised criminals who are looking for antiquities to sell on the black market may acquire archaeological reports (Ikram 2013:369). In this respect, the EAMENA/MarEA database itself could work to the disadvantage of protecting sites, although Fisher et al. (2021) have recently pointed out that there is no actual evidence for this, as looted sites are usually already known to looters. Since local governments are unlikely to have the capacity to protect every site, involvement of local communities and other stakeholders in the protection of archaeology and heritage is vital.

## Discussion and Conclusion

Throughout history, the sea has played a major role in the economy and movements of people in the Sinai. However, the coastal areas have not been comprehensively studied before, rendering our understanding of regional and maritime connectivity rather elusive. Moreover, much of the archaeological research that has been carried out dates to the period of Israeli occupation several decades ago, and/or only focuses on individual sites, ignoring their regional and landscape contexts. However, by focusing only on the Sinai as a terrestrial area, the history of the peninsula cannot be understood fully. In this paper, we have focused on the maritime character of the Sinai, creating an overview of the most occurring site types in the coastal zones. In addition, we have highlighted the most common disturbances and threats to these sites and made a first attempt to compare this to the situation in the better-researched High Mountains, which allows us to pinpoint several issues.

Currently, the biggest threat to cultural heritage is the vast expansion of urbanised areas and infrastructure, coupled with a lack of clear plans for the protection or use of most cultural heritage sites. This will inevitably lead to the degradation of the natural landscape. This is a problem in the High Mountains, but even more so in the coastal regions, where the level of institutionalised protection is lower and the pace of development much higher. Although the everyday protection of many of the sites within the St Catherine Area leaves room for improvement, basic regulations are at least in place. What is more, many of the traditional agricultural and religious sites in the High Mountains are still in use. Although this certainly causes some damage, it also protects aspects of both the tangible and intangible heritage. It is mainly in the vicinity of the town of St Catherine that there is a change in lifeways as a result of the modern tourist industry—a development that will only become stronger as a result of further proposed development—but the unsustainable character of this development is receiving unfavourable scholarly and public attention.

This is not the case for the cultural heritage on the coast, where modern construction has already replaced the traditional way of life and much of the heritage places, both along the west coast and—despite the existence of several Natural Protectorates—the east coast. The government is aware of the importance of natural and cultural heritage for the country’s tourism economy, but protection focuses on several key and accessible sites, while small and/or remote sites are ignored as a result of their lack of potential to generate tourism revenues. This is particularly problematic in the light of the noted differences in *site functions* between the coast and the High Mountains Region. An incorporation of cultural heritage within the mission statements of these protectorates, as for example suggested by Breen et al. (2021), could provide a broader inclusivity and local engagement.

In 1996, the Department of Underwater Archaeology (DUA) was founded in Egypt, which is responsible for the protection of the entire submerged heritage along the coasts of Egypt and oversees all aquatic activity along the coast and inland waters of Egypt (Abd-el-Maguid 2012). Furthermore, the Alexandria Centre for Maritime Archaeology offers academic education and training programmes for underwater archaeology and heritage management (Khalil 2008:87). However, detailed studies of underwater archaeology in the Gulfs of Suez and Aqaba are not available. Currently, the SS Thistlegorm (https://thethistlegormproject.com/), a UK ship that sank off the coast of Ras Mohammed during WWII, is one of the few extensively investigated underwater archaeological sites off the Sinai coast, and is a popular diving destination. Since diving is a major tourist attraction of the Sinai, and there are many highly experienced divers, it could be interesting to focus on training amateur divers to recognise, respect and document any remains they might encounter in order to enlarge the body of known underwater sites.

Another problem for Sinai’s heritage protection is caused by a bias towards certain time periods imposed by centralised protection strategies. The Dynastic heritage of the Nile Valley undoubtedly receives most scholarly and touristic attention; however, it is only part of Egypt’s history and is represented in the Sinai besides other extended periods, e.g. pre-historic sites. A more diverse approach to archaeology and heritage would better protect Sinai’s rich archaeological resource and could offer a chance to put in place a wider effective mutual policy and understanding between the central government and local Bedouin population. As mentioned, local interest and appreciation of archaeology and heritage are vital in protecting it, especially since information about archaeological and heritage sites is increasingly open sourced. Local involvement is not only important in order to protect sites from looting or demolition to make room for new construction: local parties should also be encouraged to participate more in research and data gathering. Our analysis clearly shows that a considerable part of the heritage sites are under threat, and that there is a need for clear management plans in order to protect sites. Which sites should be protected and how they should be protected should be decided in consultation with local stakeholders. When authorities steer away from the current situation in which the main function of archaeological sites is to serve as tourist attractions, and start seeing them as places for education and training, archaeological sites have the potential to become meaningful and relevant places for a much broader group of people than merely tourists (e.g. Ministry of State for Environmental Affairs, Egyptian Environmental Affairs Agency and Nature Conservation Sector 2006; Tuttle 2013, Holly et al. 2021; http://usaidschep.org/en). Such a localised approach would both protect archaeology and heritage and encourage a specified approach to different sites, while offering the possibility of improving the visibility of the local, regional and global connections of the Sinai. Opportunities for collaboration between the MarEA and EAMENA projects and local parties to establish such sites should be further investigated in the future.

The varied and rich archaeological record of the South Sinai coastal zone underlines its position at a crossroad and the importance of the peninsula for the long-distance movements of people and goods. The isolated and remote St Catherine Monastery is currently the focal point for archaeological interest and heritage protection in South Sinai. However, it was part of a broader network of monasteries, sites of religious importance and trade centres throughout the Sinai and beyond, linked to each other through routes that went both overland and overseas. These relations are currently undervalued, but a wider landscape-based approach can contribute substantially to our understanding of the region. This paper only presents an initial survey, and in no way claims to be a complete study of the maritime sites in the Sinai. We do nevertheless hope that it has highlighted the seriousness of the threats the coastal sites in the Sinai are facing, including from ongoing development. We also hope that we have made a compelling case for the potential of a holistic approach that would shed light on the history of the peninsula and its relation to the wider Red Sea region while encouraging public involvement to aid in their protection and increase their societal value. The (maritime) archaeology and history of South Sinai is not only a source of revenue, but part of Egypt’s Sinai identity.

## Data Availability

All data used in this paper are derived from previously published research and the EAMENA database https://database.eamena.org/en/. Analyses and/or database access is available on request.

## Footnotes

1 A heritage place can have more than one function.

2 This last category is not included in Fig. 4, because it is not among the most documented.

3 A heritage place can have more than one disturbance cause and threat category.
